# Sarcoma Metabolomics: Current Horizons and Future Perspectives

**DOI:** 10.3390/cells10061432

**Published:** 2021-06-08

**Authors:** Miguel Esperança-Martins, Isabel Fernandes, Joaquim Soares do Brito, Daniela Macedo, Hugo Vasques, Teresa Serafim, Luís Costa, Sérgio Dias

**Affiliations:** 1 Centro Hospitalar Universitário Lisboa Norte, Medical Oncology Department, Hospital Santa Maria, 1649-028 Lisboa, Portugal; fernandescristina@hotmail.com (I.F.); luiscosta.oncology@gmail.com (L.C.); 2 Vascular Biology & Cancer Microenvironment Lab, Instituto de Medicina Molecular João Lobo Antunes, Faculdade de Medicina, Universidade de Lisboa, 1649-028 Lisboa, Portugal; tserafim@medicina.ulisboa.pt (T.S.); sergiodias@medicina.ulisboa.pt (S.D.); 3 Translational Oncobiology Lab, Instituto de Medicina Molecular João Lobo Antunes, Faculdade de Medicina, Universidade de Lisboa, 1649-028 Lisboa, Portugal; 4 Faculdade de Medicina, Universidade de Lisboa, 1649-028 Lisboa, Portugal; joaquimsoaresdobrito@gmail.com (J.S.d.B.); hugovasques@sapo.pt (H.V.); 5 Centro Hospitalar Universitário Lisboa Norte, Orthopedics and Traumatology Department, Hospital Santa Maria, 1649-028 Lisboa, Portugal; 6 Medical Oncology Department, Hospital Lusíadas Lisboa, 1500-458 Lisboa, Portugal; danielavgmacedo@gmail.com; 7 General Surgery Department, Instituto Português de Oncologia de Lisboa Francisco Gentil, 1099-023 Lisboa, Portugal

**Keywords:** sarcoma, soft tissue sarcoma, bone sarcoma, gastrointestinal stromal tumor, metabolomics, metabolism

## Abstract

The vast array of metabolic adaptations that cancer cells are capable of assuming, not only support their biosynthetic activity, but also fulfill their bioenergetic demands and keep their intracellular reduction–oxidation (redox) balance. Spotlight has recently been placed on the energy metabolism reprogramming strategies employed by cancer cells to proliferate. Knowledge regarding soft tissue and bone sarcomas metabolome is relatively sparse. Further characterization of sarcoma metabolic landscape may pave the way for diagnostic refinement and new therapeutic target identification, with benefit to sarcoma patients. This review covers the state-of-the-art knowledge on cancer metabolomics and explores in detail the most recent evidence on soft tissue and bone sarcoma metabolomics.

## 1. Introduction

The knowledge regarding cancer cell properties has significantly evolved since the identification of the original hallmarks of cancer-sustaining proliferative signaling, evading growth suppressors, activating invasion and metastases, enabling replicative immortality, and inducing angiogenesis and resisting cell death [1]—and subsequent postulation of two additional ones—avoiding immune destruction and reprogramming of energy metabolism [2].

Over recent decades, the spotlight has been placed on the metabolic adaptations that cancer cells assume and in their proliferative capacity. Otto Warburg, a pioneer in cancer metabolism research, observed that, even in oxygen-rich environments, cancer cells rearrange their glucose metabolism and restrain their energy metabolism to glycolysis [3].

Recent developments have led to the comprehension of mechanisms by which specific metabolic pathways are activated, enhanced, or reprogrammed, leading to the use of accessible nutrients not only for production of metabolic precursors for cell anabolism and biosynthesis, but also to meet the bioenergetic demands required for cell perpetuation and for keeping an adequate intracellular reduction–oxidation (redox) balance [4].

This review covers the state-of-the-art knowledge on cancer metabolomics and explores in detail the most recent evidence on soft tissue and bone sarcoma metabolomics, identifying potential biomarkers and therapeutic targets that may modulate these metabolic pathways.

## 2. Cancer Metabolic Fingerprints

Neoplastic clones have the capability to adapt their metabolic activity to support the various tumorigenesis stages. These adaptations cover all phases of cell–metabolite interactions, influencing the metabolite inflow and increasing cell’s ability to obtain the necessary nutrients, shaping the way nutrients are selectively allocated to metabolic pathways that fuel cellular tumorigenic adjustments, and providing long-ranging effects on cellular fate, amid which are changes in differentiation of both cancer cells and tumor microenvironment elements [5]. Metabolic reprogramming can be defined as the increase or suppression of standard metabolic pathways activity in cancer cells as a product of tumorigenic mutations [6]. Oncometabolites are the metabolites whose quantity is markedly increased in cancer cells, with an existing link either between their accumulation and a particular mutation in the tumor, or between their presence and cancer development [6].

**Figure 1:**
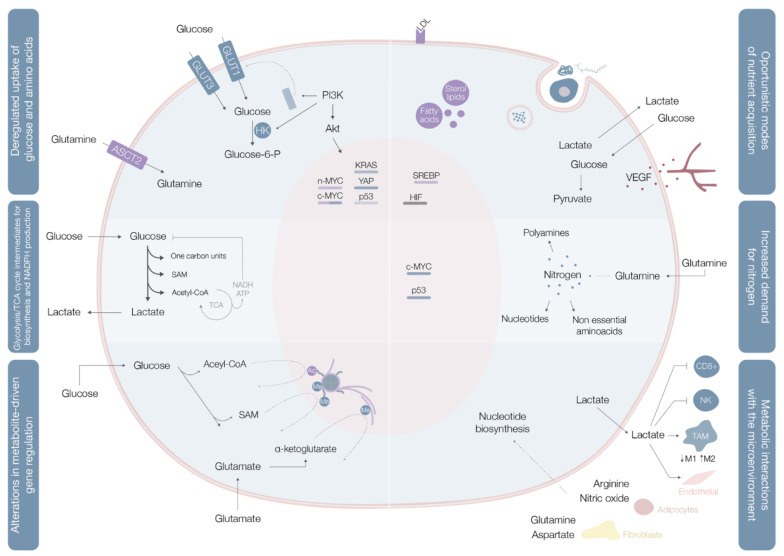
Cancer metabolic fingerprints. GLUT1—Glucose transporter 1; GLUT3—Glucose transporter 3; ASCT2—Alanine, serine, cysteine transporter 2; PI3K—Phosphoinositide 3-kinase; Akt—Protein kinase B; HK—Hexokinase; Glucose-6-P—Glucose-6-phosphate dehydrogenase; LDL—Low-density lipoprotein; VEGF—Vascular endothelial growth factor; SREBP—Sterol regulatory element-binding proteins; HIF—Hypoxia inducible-factors; SAM—S-Adenosyl methionine; Acetyl-CoA—Acetyl coenzyme A; NADH—Nicotinamide adenine dinucleotide; ATP—Adenosine triphosphate; TCA—Tricarboxylic acid cycle; NK—Natural killer cells; TAM—Tumor-associated macrophages.

These rearrangements provide cancer cells with the power to fulfill their biosynthetic, bioenergetic and redox balance needs, and include three layers of cell-metabolite interactions (oncogene-directed nutrient uptake, intracellular metabolism reprogramming, and metabolite-directed changes in cell behavior/function). This metabolic reshaping may be summarized in six hallmarks of cancer metabolism: deregulated glucose and amino acid uptake, use of opportunistic modes of nutrient acquisition, use of glycolysis/tricarboxylic acid (TCA) cycle intermediates for biosynthesis and NADPH production, increased nitrogen demand, alterations in metabolite-driven gene regulation, and metabolic interactions with the tumor microenvironment [5] (Figure 1).

### 2.1. Deregulated Glucose and Amino Acid Uptake

Cancer cells import or use different types of nutritional fuels to fulfill their core metabolic functions [7]. Glucose and glutamine are the two most prominent nutrients, and the main sources for maintenance of diverse carbon intermediates pools used as elementary units for assembly of diverse macromolecules, combustibles for adenosine triphosphate (ATP) generation, and cellular redox capacity enhancers [5].

Aerobic glycolysis (Warburg effect), the process of importing glucose and exporting carbon as lactate even in oxygen-rich environments, is the most widely explored metabolic pathway in cancer cells. When in cytosol, glucose may be used as a substrate in glycolysis (where the resulting pyruvate contributes to acetyl-CoA synthesis, crucial for the production of fatty acids, lipids and cholesterol, and non-essential amino acids aspartate and asparaginase [4]), in the hexosamine synthesis pathway (HSP), pentose phosphate pathway (PPP) [4], and serine biosynthesis pathway (SBP) [8]. Glucose catabolism is used by cancer cells as a way of generating precursors and intermediates for many other metabolic pathways.

In benign cells, nutrient assimilation is regulated by growth factor signaling and cell interactions with the extracellular matrix [5]. Cancer cells carry a panoply of mutations that bestow them with a significant degree of independence from these external requirements [5]. Different mutations result in constitutive glucose uptake and metabolic adaptations [5]. Mutations in c-MYC, KRAS, and YAP oncogenes upregulate transmembrane protein glucose transporter (GLUT) 1 expression, while overexpression of YAP and loss-of-function mutations in p53 augment GLUT3 expression, enhancing glucose entrance into the cell [8] (Figure 1, Table 1). In parallel, the phosphoinositide 3-kinase/protein kinase B (PI3K/Akt) pathway is typically hyperactivated and acts as a master regulator of glucose uptake—by promoting GLUT1 mRNA expression and GLUT1 protein translocation from the inner membranes to the cell surface [5] on the one hand, and of phosphorylation—upregulating hexokinase (HK) 2 activity and trapping glucose inside the cell [8]—on the other (Figure 1, Table 1). HK generates glucose-6-phosphate dehydrogenase (G6PD), a PPP starter, which represents a pivotal pathway for production of nicotinamide adenine dinucleotide phosphate (NADPH), crucial for fatty acid synthesis and glutathione regeneration, for keeping the redox equilibrium, and for production of ribulose-5-phosphate, fundamental for nucleotide synthesis [8]. Different oncogenes enhance PPP activity, with overactive PI3K/Akt and mTORC1 signals augmenting the expression of rate-limiting enzymes in this pathway [8]. Akt hyperactivation promotes increased transketolase enzyme activity, c-Myc stimulates PPP inflow, and p53 loss-of-function mutations increase PPP activity [8].

Glutamine is the most copious plasmatic amino acid, providing critical elements for cell proliferation, like carbon and nitrogen [8]. Glutamine influx into the cytoplasm depends on alanine, serine, cysteine transporter 2 (ASCT2) glutamine transporter, the expression of which is upregulated by c-MYC and n-MYC via activating transcription factor 4 (ATF4) in neuroblastoma, induced by mTORC1, and regulated by microenvironment factors, such as IL-4 and lactate [8] (Figure 1, Table 1). Glutamine can also be imported by micropinocytosis in Ras-mutated cancer cells [8] (Table 1). When inside the cell, glutamine may, as a nitrogen donor, fuel amino acid and nucleotide biosynthesis or, as a carbon donor, fuel fatty acid synthesis [8]. Glutamine may also be a source of nicotinamide adenine dinucleotide (NADH) and flavin adenine dinucleotide (FADH_2_) conferring reducing power to deal with reactive oxygen species [8].

### 2.2. Use of Opportunistic Modes of Nutrient Acquisition

Cancer cells have the ability to thrive in nutrient-deprived environments, carrying specific mutations that enable them to use unorthodox methods of nutrient acquisition [5]. They have the capacity of finely monitoring the accessible extrinsic nutrients, whose availability oscillates throughout different oncogenesis phases, to orchestrate appropriate metabolic responses [9]. The proficiency of cancer cells in these processes may be achieved by induction of gene expression programs that modulate the activity of nutrient transporters and specific metabolic enzymes [9]. For instance, hypoxia triggers the expression of transcription factors, called hypoxia-inducible factors (HIF), which stimulate glucose uptake, lactate export, glycolysis, and angiogenesis [9] (Figure 1, Table 1). Moreover, cholesterol depletion induces activation of sterol regulatory element-binding proteins (SREBP), a family of transcription factors that stimulate the expression of almost every single enzyme required for de novo synthesis of fatty acid and sterol lipids, also leading to augmented low-density lipoprotein (LDL) receptor expression and enhanced NADPH production [9] (Figure 1, Table 1). In turn, amino acid deprivation leads to general control nonderepressible 2 (GCN2) kinase activation, resulting in selective translation of mRNAs like ATF4, which ultimately promote the transcription of amino acid transporters and enzymes involved in the generation of non-essential amino acids [9] (Figure 1, Table 1).

Besides the above-mentioned mechanisms, cancer cells have developed strategies to capture extracellular macromolecules. Mutant Ras or c-Src cancer cells are capable of recovering free amino acids through lysosomal digestion of extracellular proteins [5]. Neoplastic clones are able to capture extracellular macromolecules through macropinocytosis, a process stimulated and driven by Ras and c-Src actin cytoskeleton remodeling, in which significant quantities of extrinsic fluid are engulfed by macropinosomes that are transported into the cell, where they fuse with lysosomes, allowing the proteolytic digestion of immersed proteins [5]. Furthermore, amino acids can be retrieved from the envelopment and degradation of entire living cells via entosis, as well as from phagocytic digestion of apoptotic cellular corpses [5] (Figure 1, Table 1). In parallel, emergence of hypoxic areas compromises biosynthetic reactions that demand oxygen as an electron acceptor, leading for example, to unsaturated fatty acid species deficit [5]. Hypoxic cancer cells can import unsaturated fatty acids from the circumambient in the form of single acyl chain-containing lysophospholipids, increase the extrinsic liberation of free fatty acids from more complex lipid species, and induce the release of stored lipids from surrounding neighbor normal cells [5]. Under extreme conditions of prolonged periods of extracellular nutrient absence, cancer cells may initiate a self-catabolic process of macroautophagy, with sequestration and lysosomal digestion of cytosolic macromolecules and organelles, allowing the recycling of these cellular components into nutrients that may be catabolized for energy production or used in biosynthesis of new macromolecules [5,9] (Figure 1, Table 1). Macroautophagy plays key roles in distinct processes, like tumorigenesis, microenvironment interaction, metastases, and drug resistance [10]. Treatment with tyrosine kinase inhibitors (TKI) (lapatinib, used for breast cancer treatment, is a good example) induce cancer cells autophagy and apoptosis [11]. Nevertheless, resistance to TKI (such as lapatinib) may be verified, with autophagosome and autolysosome proteins changes probably contributing to those mechanisms of resistance [11].

Keeping in mind that G6PD is a crucial enzyme of the PPP, the main source of NADPH (that antagonizes oxidative stress generated in highly metabolizing neoplastic cells), it is interesting to note that G6PD inhibition induces reactive oxygen species production and enhances endoplasmic reticulum (ER) stress [11]. These biological effects are related to augmented autophagic flux [11]. Mele et al. observed that G6PD blockade in breast cancer cells caused a congruous increase of autophagosomes formation independently from the mammalian target of rapamycin (mTOR) status, synergistically augmenting the lapatinib-induced cytotoxic effect on cancer cells [11].

### 2.3. Use of Glycolysis/TCA Cycle Intermediates for Biosynthesis and NADPH Production

Glycolysis and the tricarboxylic acid (TCA) cycle are used by proliferating cells as production lines of metabolic intermediates for different biosynthetic processes. Cancer cell subpopulations are heterogenous regarding nutrient requirements and metabolic adaptations to accomplish biosynthetic and bioenergetic purposes.

Contrarily to quiescent cells, in which glucose is directed for mitochondrial acetyl-CoA and ATP production, proliferating cells use reduced carbon for the biosynthesis of a broad plethora of biomolecules [5] (Figure 1). To accomplish this, cells must convert acquired nutrients into different pools of structural intermediates, including cytoplasmic acetyl-CoA, one-carbon carrying folate cycle units, S-adenosylmethionine (SAM), and an array of glycolytic and TCA cycle intermediates [5]. Many of these biosynthetic reactions are intrinsically reductive and require a reductive power source, typically NADPH [5].

Proliferating cells use the Warburg effect in a regulated way during periods of augmented biosynthetic requirement [5]. They convert excess pyruvate to lactate instead of transporting it to the mitochondria [5]. These cells have only a small ATP consumption increment in relation to their needs for precursor molecules and reducing equivalents in the form of NADPH [5]. Glucose catabolic processing is a strong supplier of these precursors and reducing equivalents, while the NADH- and ATP-producing TCA cycle represent the major negative regulator of glucose metabolism [5]. Transforming excess pyruvate in lactate prevents accretion of NADH and diminishes ATP production, avoiding glucose metabolism feedback repression by excessive mitochondrial ATP generation [5].

Glycolysis and the TCA cycle generate intermediates that can be diverted into branching pathways for production of different biosynthetic precursors, with overexpression of key enzymes of different pathways in distinct cancer cells [5]. Diverse oncogenes orchestrate these adaptations, with c-MYC and β-catenin/TCF signaling coordinately shaping PDK1, lactate dehydrogenase A (LDH-A), monocarboxylate transporter (MCT1), and HIF1α expression [5] (Table 1).

Quiescent tumor cell subpopulations are significantly less glycolytic and show higher dependence on oxidative phosphorylation (OXPHOS) with elevated expression of mitochondrial respiratory components and using carbon predominantly for bioenergetic purposes [5].

### 2.4. Increased Demand for Nitrogen

Growth signaling increases the cellular demand for reduced nitrogen [5]. A proliferating cell must synthesize different nitrogen-containing molecules, such as nucleotides, nonessential amino acids, and polyamines [5].

Glutamine contains two reduced nitrogen atoms, representing the main way for reduced nitrogen trafficking between cells. Its amide group is a nitrogen donor for purine and pyrimidine base synthesis [5]. In parallel, assembly of both pyrimidine and purine rings requires aspartate, originated from transamination of oxaloacetate and glutamic acid, both glutamine catabolites [5]. Glutamine levels are rate-limiting for cell cycle progression and deprivation of these levels may lead to cell cycle arrest in S phase [5].

Not only does c-MYC promote cellular glutamine uptake, it also regulates nucleotide biosynthesis by upregulating the expression of a vast array of enzymes with specific roles in the process [5] (Table 1, Figure 1).

Glutamine can be directly deaminated to glutamate by glutaminase, an enzyme often upregulated in neoplastic clones in a c-MYC-dependent manner [5]. Glutamate may also be a nitrogen donor for production of nonessential amino acids via transamination [5]. Conversely, asparagine biosynthesis from aspartate, catalyzed by asparagine synthetase, uses the amide nitrogen of glutamine [5]. Asparagine plays a fundamental regulatory role in glutamine deprivation conditions, with asparagine synthetase being frequently upregulated in tumors [5] (Table 1).

Most proliferating cells require an exogenous glutamine source, but particular cell types show the ability to proliferate in its absence, suggesting the occurrence of intracellular de novo production [5]. Glutamine synthetase has been found to be overexpressed in some cancers, with its mechanism of action still requiring clarification [5] (Table 1).

### 2.5. Alterations in Metabolite-Driven Gene Regulation

Metabolic reprogramming can support the transformation of benign into malignant cells, a process conducted by aberrantly activated growth and survival signals [5,12]. The metabolic matrix is not merely a passive recipient of growth signals, but also transmits information related to cell metabolic state and regulatory enzymes, including those that mediate the deposition and removal of epigenetic marks from chromatin [5]. Oncogenic mutations may affect genes encoding all types of epigenetic machinery, including histones, chromatin modifiers and remodelers, and epigenetic readers [12]. Concurrently, metabolic genes involved in production of chromatin-modifying metabolites are also frequently mutated in cancer [12].

Histone acetylation can be tuned in cancer cells by acetyl-CoA titer modulation, since cytoplasmic acetyl-CoA is the obligate substrate for enzymes that acetylate histones and other proteins [5,12]. Placement of acetyl marks on histones leads to increased genomic DNA accessibility, enabling assembly of transcriptional complexes. This process has a quick turnover rate, as histone acetylation is extraordinarily sensitive to any fluctuations in the cellular nutritional and signaling status [5]. Oscillations in glucose availability and oncogenic pathway activation promote total histone acetylation enhancement, leading to increased and broader gene expression [5] (Figure 1, Table 1).

Deposition of methyl marks on histone tails and the processes of cytosine methylation on DNA and adenosine methylation on mRNA, use SAM as the methyl group donor [5] (Figure 1). SAM results from the one-carbon metabolic pathway and is powered by serine catabolism [5]. Histone and DNA methylation are sensitive to SAM concentrations shifts [5].

Different cell posttranscriptional changes are mediated by different α-ketoglutarate-dependent dioxygenases [5]. Among these are the TET family of DNA demethylases, the Jumonji C (JmjC) family of histone demethylases, mRNA demethylases FTO and ALKBH5, and a family of prolyl hydroxylase enzymes (responsible for regulating HIF1α levels according to oxygen levels and oxidative stress) [5]. Intracellular levels of α-ketoglutarate influence activity of these enzymes [5]. These dioxygenases are also susceptible to inhibition by their reaction product, succinate, as well as by fumarate, the succinate degradation downstream product in the TCA cycle [5]. Genes encoding the succinate dehydrogenase (SDH) metabolic enzyme frequently show loss-of-function mutations in different tumors, leading to succinate accumulation, inhibition of JmjC-domain-containing demethylases and, ultimately, to genome-wide DNA and histone hypermethylation [5,12] (Table 1). Similarly, loss-of-function mutations of fumarate hydratase (FH) are also seen in some tumors, leading to fumarate accumulation, inhibition of TETs and genome-wide DNA, and histone hypermethylation [5,12] (Table 1). DNA and histone hypermethylation provided by SDH and FH mutations enables oncogenic promoter-enhancer interactions, induces epithelial-to-mesenchymal transition, and disrupts DNA repair mechanisms [12] (Table 1). Mutations in isocitrate dehydrogenase (IDH1) and isocitrate dehydrogenase 2 (IDH2) represent a different group of cancer-related genetic alterations responsible for regulating the activity of α-ketoglutarate-dependent-dioxygenases [5]. These mutations have been identified in chondrosarcomas, among other cancer types [5]. Contrarily to wild-type IDH1/2, which convert the TCA cycle metabolite isocitrate to α-ketoglutarate, mutant IDH 1/2 use α-ketoglutarate as a substrate catalyzing its conversion to D-enantiomer of 2-hydroxyglutarate (2-HG) [5]. 2-HG, structurally similar to α-ketoglutarate, is a competitive inhibitor of α-ketoglutarate-dependent-dioxygenases [5]. IDH-driven cancers have a prominent CpG island hypermethylation similar to the hypermethylation phenotype seen on SDH- and FH-deficient cancers [5]. Therefore, IDH1 and IDH2 mutant genes can lead to DNA and histone hypermethylation though 2-HG accumulation, with subsequent downregulation of genes associated with tumor suppression and cellular differentiation blockade [12] (Table 1).

Removal of acetyl and methyl marks is similarly driven by the cellular metabolic state, with sirtuins coordinating posttranslational and epigenetic changes leading to energy conservation [5,12].

### 2.6. Metabolic Interactions with the Microenvironment

The methods by which cancer cells modulate their microenvironment to assist tumor growth and dissemination remain largely unexplored, even though it is clear that they encompass diverse strategies, including growth factor secretion and extracellular matrix and cell–cell interaction adaptations [5].

The tumor microenvironment (TME) is composed of various cell types organized in a unique metabolic landscape [13]. Blood supply and stromal and immune cells modulate tumor growth and development [13]. TME is typically hypoxic, acidic, nutrient-deprived, and electrolyte imbalanced, displaying elevated oxidative stress levels as a product of high metabolic activity of cancer cells, abnormal blood flow, and important inflammation [13]. The metabolic niche within the TME is shaped by four regulation tiers: (1) intrinsic tumor cell metabolism; (2) competition and crosstalk between cell types; (3) tumor location and heterogeneity; and (4) whole-body metabolic homeostasis [13].

The intrinsic tumor cell metabolism imprint on the TME metabolic niche has already been explored.

The TME is home to a complex immune cell environment [13]. Natural killer (NK) and CD8+ T cells are labelled as cytotoxic lymphocytes, while CD4+ T (like TH1 or TH17) cells support or repress (Treg cells) the activity of other immune cells [13]. Metabolites and metabolic pathways can regulate T-cell function, fate, and differentiation [13]. Activation of both T and cancer cells relies on glucose metabolism and limiting glucose availability leads to competition between both cell types (low CD8+ T-cell infiltration in glycolytic tumors supports this idea) [13]. Low glucose levels impair T cell function and proliferation by decreasing mTOR activity [13] (Table 1). Apart from glucose restriction, extracellular lactate accumulation also leads to in vitro and in vivo CD8+ T-cell and NKcell infiltration impairment [13] (Table 1, Figure 1). Nonetheless, T cells display a certain degree of metabolic flexibility. For instance, when in glucose deprived TMEs, CD8+ T cells are able to upregulate fatty acid catabolism to generate energy [13].

Amino acids like glutamine, L-arginine, and methionine have also shown effects on function and differentiation of different T cells [13].

Tumor-associated macrophages (TAM) may present different phenotypes. While M1 macrophages show a proinflammatory (antitumoral) profile, M2 counterparts display an anti-inflammatory (protumoral) profile, with each state being portrayed by different markers and gene expression [13]. TAM polarization may be driven by different soluble factors secreted by neighboring cells, genetic background, and cellular metabolism [13]. TAMs, like T cells, compete with adjacent cells for glucose [13]. Hypoxic TAMs show high expression of the negative mTOR regulator REDD1 and diminished glycolysis [13]. Culturing human blood monocytes with media derived from pancreatic ductal adenocarcinoma (PDAC) cell lines leads to development of highly glycolytic TAMs with increased metastatic potential [13]. Lactate promotes M2 polarization by inducing VEGF production and promotes epigenetic alterations in bacterially challenged M1 macrophages [13] (Table 1, Figure 1). Glutamine metabolism is associated with protumoral TAMs polarization through production of α-ketoglutarate, an inducer of fatty acid oxidation and epigenetic upregulation of M2 genes [13].

Stromal cells may also regulate tumor cell behavior, by contributing to ECM remodeling and cancer cell migration, invasion, and immunosurveillance escape [13]. These cells derive from distinct cell types, producing cancer-associated fibroblasts (CAF), adipocytes or endothelial cells [13]. The metabolic crosstalk between CAFs and cancer clones is frequently mentioned as the “reverse Warburg effect”, since metabolites secreted from CAF glycolysis are used as fuel for adjacent cancer cells [13] (Table 1). Glutamine anabolic metabolism is increased in CAF, with glutamine being secreted and used by neoplastic clones to sustain nucleotide generation and OXPHOS [13] (Table 1, Figure 1). CAF also secrete aspartate, which supports nucleotide biosynthesis and cell proliferation in multiple cancers [13] (Figure 1). Oppositely, glutamate secreted by cancer cells may feed glutathione (GSH) production keeping redox balance and ECM remodeling in CAF [13]. CAF also shape cancer metabolism through direct cancer cell support, since CAF-derived exosomes supply cancer cells with amino acids, lipids and TCA intermediates replenishing its central carbon metabolism [13].

Different tissues and organs are defined by specific epigenetic modulation, gene expression, proteomes, and metabolomes [13]. The contrast between different tissue metabolisms suggests the possibility of cancers arising in different organs with different metabolite landscapes [13]. Recent evidence suggests that the metabolic gene expression program remains more similar to that of the original tissue where cancer is located than that of similar cancers in distinct organs [13]. Oncoproteins, such as SDH and FH, induce tumorigenesis only in specific tissues, supporting the idea that the tissue of origin defines mutational penetrance [13]. The metabolic phenotype of the neoplastic clone can evolve to more efficiently use available local metabolites [13].

The influence of local microenvironment composition on tumor metabolism is more evident when comparing primary and secondary lesions of the same tumor of origin [13]. For instance, primary breast tumors depend on glutamine anaplerosis, while lung metastases use the pyruvate-rich lung environment to increase PC activity, inducing proliferation of established secondary lesions, remodeling ECM, and stimulating the transition to the macrometastatic stage [13]. Within the same organ, a tumor can develop in different topographies and thus adapt to different environments [13]. The degree of perfusion, different tissue function, and cell-type composition all play a role in this metabolic spatial heterogeneity [13]. In some cancer cell types, a vigorous correlation was found between glycolysis and mitochondrial metabolism and local oxygen availability [13] (Table 1). Perivascular tumor cells display extremely high mTOR-dependent anabolic metabolism and increased tumorigenesis in mouse glioblastoma xenografts [13]. Solid neoplasms themselves are metabolically heterogeneous, with cancer cells in well perfused areas consuming glucose and sustaining glycolysis and OXPHOS, while cells on poorly perfused areas rely on other carbon sources [13]. Solid tumors contain glutamine (besides aspartate, asparagine, and serine) depleted core regions, a phenomenon that may induce hypermethylation and dedifferentiation [13].

The TME is also shaped by systemic, organismal metabolism, which is a product of the overall metabolic state of an individual, and by environmental factors such as diet [13]. Dietary interventions and hormonal modulation may influence local metabolism [13]. Modulation of the amino acid diet composition has been investigated in cancer progression and treatment settings [13]. Global caloric restriction diminishes lipid availability in plasma and tumor interstitial fluid as well as remodels PDAC lipid metabolism, inhibiting stearoyl-CoA desaturase activity and constraining PDAC progression by toxic saturated lipid accumulation [13]. Dietary modification may be synergistically combined with pharmacological approaches [13]. PI3K inhibition leads to systemic glucose–insulin feedback that might reactivate the PI3K-mTOR signaling axis in tumors [13]. The efficacy of PI3K inhibitors is frankly augmented by ketogenic diet treatment aimed at inhibiting this feedback [13].

## 3. Sarcoma Metabolomics

Sarcomas are rare and heterogenous neoplasms of mesenchymal origin, accounting for 1% of adult and 15% of pediatric cancers and comprising almost 100 histological subtypes [14]. The greater part (≈75%) of sarcomas develops from soft tissues, while a smaller percentage develops from bone (≈10%) [15].

Approximately 35–45% of sarcoma patients present distant recurrence, even after standard systemic treatment [16]. The 5-year survival rate of patients with metastatic disease is only 16%, with chemotherapy virtually representing the single systemic treatment option [16].

Several molecular alterations associated with different sarcoma types have shown diagnostic and prognostic value. Conversely, the number of genetic and molecular changes with disease monitoring and treatment utility is very small [17,18].

Sarcomas, like other tumors, display abnormal metabolic activity patterns, but these are far from being extensively explored or correlated with specific gene mutations.

Most studies on sarcoma metabolomics have used cell lines [19], as their metabolome status is much easier to freeze.

Deepening sarcoma metabolomic and microenvironmental knowledge may allow to identify new potential diagnostic and therapeutic targets, improving patients’ survival and quality of life. Specific characteristics of soft tissue and bone sarcoma metabolomics will be further explored.

### 3.1. Soft Tissue Sarcoma Metabolomics

Detailed data regarding soft tissue sarcoma (STS) metabolome is relatively sparse. Different oncogenes and tumor suppressors implicated in metabolic pathway regulation are mutated in sarcomas, like PIK3CA, TP53, and NF1 [20]. Furthermore, hypoxic tumor microenvironments, characteristic of sarcomas, modify metabolism and correlate with worse prognosis [21].

Recent evidence has emerged identifying the prognostic value and potential therapeutic usefulness of some STS metabolites.

Lou et al. used mass spectroscopy imaging to identify prognostic metabolite biomarkers in high-grade sarcomas using 33 samples, including leiomyosarcomas (LMS), myxofibrosarcomas, and undifferentiated pleomorphic sarcomas (UPS) [19]. The authors identified carnitine (poor metastases-free survival in myxofibrosarcoma patients) and inositol (1,2-) cyclic phosphate (poor overall survival in STS patients) as potential generic prognostic biomarkers [19].

Miolo et al. enrolled 24 patients with metastatic STS scheduled for treatment with trabectedin in a metabolomic study aimed to enhance overall survival prediction in patients [22]. The authors showed that levels of the proteinogenic amino acid citrulline and of the essential amino acid histidine significantly correlated with overall survival in STS [22]. A risk prediction model integrating metabolomics and clinical data—including citrulline and hemoglobin levels and patient performance status—allowed distinction between a high-risk group of patients with low median overall survival of 2.1 months and a low-to-moderate risk group of patients with a median overall survival of 19.1 months (*p* < 0.0001) [22]. Citrulline, an amino acid that plays an important role in arginine metabolism, represents an important metabolic signature that may contribute to explain the high inter-patient overall survival variability in STS patients [22]. The risk prediction model may represent a new prognostic tool for the early classification of metastatic STS patients, according to their overall survival expectancy [22].

**Figure 2:**
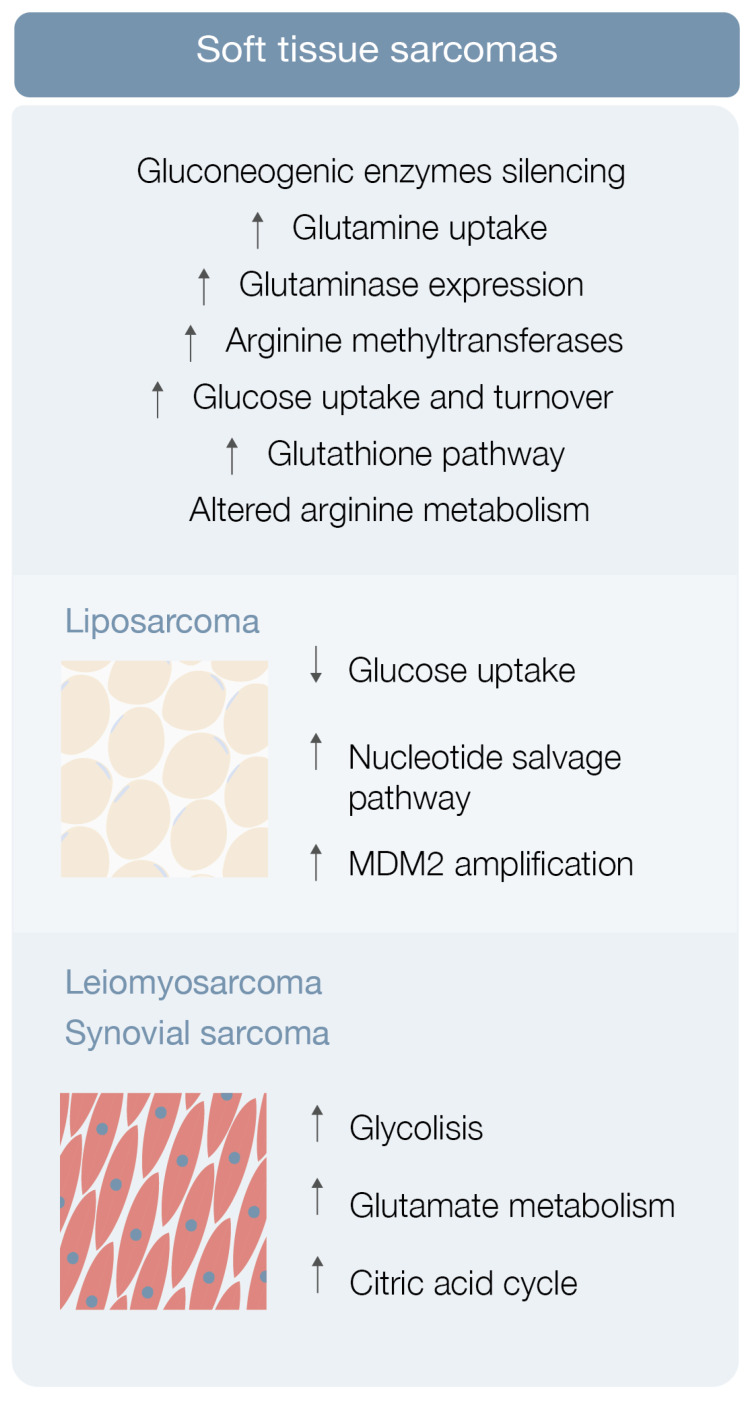
Soft tissue sarcoma metabolic hallmarks.

Sarcoma cells display elevated glucose uptake and turnover [23] (Figure 2). Gluconeogenesis counterbalances glycolysis, and gluconeogenic enzymes may be key features for tumor cell growth regulation [23]. Fructose-1-6-biphosphatase 2 (FBP2) is one of these gluconeogenic enzymes and Huangyang et al. showed that its expression is silenced in a vast array of STS subtypes [23] (Figure 2). This group also demonstrated that FBP2 re-expression suppresses sarcoma growth, by antagonizing the Warburg effect and restraining mitochondrial biogenesis and respiration, representing a potential therapeutic target [23].

Increased glutamine uptake is an also well-known metabolic adaptation of cancer cells (Figure 2). Lee et al. used autochthonous UPS murine models and human fibrosarcoma and LMS cell lines in a metabolomic analysis, and demonstrated that these specific STS types have significant glutamine dependency as well as display high glutaminase expression [24] (Figure 2). STS subtypes expressing high glutaminase levels and relying on high glutamine availability are particularly sensitive to glutamine starvation. Glutamine is mainly produced by surrounding muscle tissues, making limb sarcomas dependent on exogenous sources (like UPS and FMS) more sensible to glutamine deprivation, contrarily to STS subtypes not expressing glutaminase, as liposarcoma [24]. Telaglenastat (CB-839), a potent glutaminase inhibitor, blunted in vivo UPS growth and proliferation in tumor-bearing mice [24]. These results suggest that glutamine metabolism drives sarcomagenesis, with CB-839 showing promising therapeutic potential [24].

Finally, arginine metabolism reshaping, including protein arginine methyltransferase overexpression, may also play a role in sarcomagenesis [25] (Figure 2). Use of an arginine methyltransferase inhibitor showed antitumor effects on mouse sarcoma in 180 cells and displayed encouraging therapeutic utility [25].

Particular features of liposarcoma, LMS, and synovial sarcoma metabolic landscape will be further explored.

#### 3.1.1. Liposarcoma

Liposarcoma is the most common STS, representing around 20% of all sarcomas [26]. Patients with high-grade or unresectable liposarcoma have poor prognosis, although surgery and chemotherapy, specifically with anthracyclines, ifosfamide, antimitotic docetaxel, and antimetabolites gemcitabine, seem helpful [27,28].

Braas et al. reported a new diagnostic biomarker and treatment target retrieved from a metabolomic study [29]. In the study, metabolomic analysis of three liposarcoma cell lines frequently exhibiting low glucose uptake by positron emission tomography (PET) was performed [26,29]. Ten metabolites, comprising ascorbic acid, cholesteryl sulfate, five amino acids and amino acid precursors, and three nucleosides (cytidine, thymidine, and uridine) were consistently consumed, supporting the hypothesis that liposarcoma cells have nucleoside salvage pathway activity responsible for increasing nucleoside uptake and conversion to nucleotide triphosphates that can be incorporated into DNA [26,29] (Figure 2). This salvage pathway was discovered to be dependent on deoxycytidine kinase (dCK) in vitro and could be visualized by PET in vivo with 1-(2′-deoxy-2′-[18F] fluoroarabinofuranosyl) cytosine (FAC) [26,29]. Nevertheless, these cells were not dependent on this pathway for proliferation and survival [26,29]. Concomitantly, these liposarcoma cell lines and xenograft tumors were clearly sensitive to gemcitabine (a chemotherapeutic nucleoside analogue prodrug metabolized in a similar way as FAC) [26,29]. In other in vitro and in vivo studies, gemcitabine displayed a cytotoxic effect on liposarcoma cells exhibiting nucleotide salvage pathway activity and this gemcitabine sensitivity was dependent on dCK expression [26,29]. This body of evidence suggests that liposarcoma patients with active nucleotide salvage activity or dCK expression may be analyzed by PET imaging with [18F]-FAC and treated with gemcitabine [26,29].

Dedifferentiated liposarcoma is one of the most aggressive types of liposarcoma, characteristically associated with amplification of MDM2, a TP53 tumor suppressor inhibitor [30] (Figure 2). Individuals with greater MDM2 amplification show less chemotherapy sensitivity and worse outcomes than patients with lower MDM2 amplification [30]. A study was conducted to demonstrate that MDM2 amplification levels could be associated with changes in these tumors’ metabolism, in which six patient-derived dedifferentiated liposarcoma models were put through a comprehensive metabolomic and lipidomic analysis to ascertain associations with MDM2 amplification and response to metabolic disorders [30]. Comparison of the metabolomic profile of upper and lower MDM2 amplification cells revealed differences in a total of 17 metabolites, including ceramides, glycosylated ceramides, and sphingomyelin [30]. Lipid metabolism disturbance by statin administration led to a chemosensitive phenotype exclusively in lower MDM2 cell lines, raising the hypothesis that lipid metabolism may be a contributor to the more aggressive nature of upper MDM2-expressing tumors [30]. This and other studies greatly highlight the importance, the significance, and the contribution that lipids may play in the metabolic landscape of soft tissue sarcomas, by providing alternative energy sources and building blocks for membrane synthesis (among other properties), affecting the metabolism of STS cells, and inducing metabolic reprogramming favoring the expansion of well adapted tumor cell clones.

#### 3.1.2. Leiomyosarcoma, Synovial Sarcoma and Others STS

Leiomyosarcoma and synovial sarcoma are rare sarcomas, accounting for 5–10% of all STS [26]. Not much is known about the metabolic environment of these STSs besides the previously explored utility of carnitine in myxofibrosarcoma patients (levels correlate with poor metastasis-free survival) and inositol (1,2-) cyclic phosphate in STS patients (levels correlate with poor overall survival) [19], the role of citrulline as a prognostic marker in metastatic STS patients [22], the blunted expression of FBP2 in STS and possible utility of its re-expression induction as a therapeutic strategy [23], the high expression of glutaminases and the potential usefulness of telaglenastat on restraining sarcoma growth [24], and the overexpression of protein arginine methyltransferases and potential use of their inhibitors in sarcomagenesis control [25].

Leiomyosarcoma, synovial sarcoma, and liposarcoma metabolomic assessment robustly detected 119 metabolites [31]. Eight of these showed significantly different levels in sarcoma samples (versus normal controls), including carbamoyl phosphate, CMP, ribose-phosphate, cytosine, cyclic-AMP, DL-pipecolic acid, Ng, and NG-dimethyl-L-arginine [31]. Pathway enrichment analysis revealed that a significant number of pathways were enriched in all 119 metabolites, comprising glycolysis, glutamate metabolism, and the citric acid cycle [31] (Figure 2). Hence, STS metabolomics data may be used as diagnostic biomarkers for STS subtypes [26,31].

A specific mention should also be made to rhabdomyosarcoma, a myogenic tumor (characterized by its incapacity to leave the proliferative myoblast-like state) labelled as the most frequent STS affecting children and adolescents [32]. Genomic and transcriptomic portrayal involves either chromosomal translocation leading to the generation of the oncogenic fusion transcription factor PAX 3/7-FOXO1 or mutations in receptor tyrosine kinase/RAS pathways [33]. Specifically, PAX3-FOXO1 not only plays chromatin-level roles establishing a myoblastic super enhancer landscape, but also drives the transcription of both the glucose transporter 4 (GLUT4) gene (augmenting glucose uptake by cancer cells) and carnitine palmitoyltransferase (CPT1A) (external mitochondrial enzyme responsible for acyl carnitines production) gene (facilitating lipid degradation and subsequently providing cancer cells the energy necessary to migrate and metastasize) [32].

### 3.2. Bone Sarcoma Metabolomics

Evidence regarding bone sarcomas metabolism is scarce. The particularities of bone metabolism and the rarity of bone sarcomas account for the still important evidence gaps that need to be tackled.

#### 3.2.1. Osteosarcoma

Osteosarcoma (OS) is the most common primary malignant bone tumor in children and adolescents, maintaining a steady overall prognosis despite introduction of new chemotherapy strategies and significant advances in surgical resection, with complex reconstruction and limb salvage procedures [34]. Patients with localized disease have a 60% overall survival rate and those with metastatic or relapsed disease after initial treatment have very dismal prognosis [34]. This highlights the urgency for a better understanding of the disease nature, with metabolomics representing a promising and sparingly explored path to walk through.

**Figure 3:**
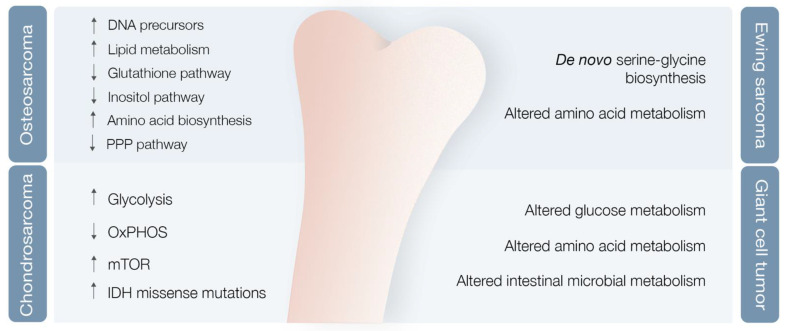
Bone sarcoma metabolic hallmarks. PPP—Pentose phosphate pathway; OxPHOS—Oxidative phosphorylation; mTOR—mechanistic target of rapamycin; IDH—Isocitrate dehydrogenase.

The OS metabolic profile remains incomplete [35]. Researchers used mouse OS models to examine different metabolic markers and found correlations between metabolic adaptations, tumor progression and metastases [36]. Various markers were differentially expressed after lung metastases development compared with nonmetastatic state, with levels of cholesterol and fatty acids, such as elaidic acid, octadecanoic acid, and decosahexaenoic acid, clearly increased, and other metabolic markers clearly decreased [36] (Figure 3). OS likely undergoes an overall metabolic decrease throughout the pulmonary metastases period, as a consequence of hypoxia and shift from consumption of amino acids and carbohydrates to lipids [36]. Pulmonary metastatic nodules were shown to be less likely formed after incorporation of synvinolin, a cholesterol synthesis-inhibiting drug [37]. Concomitantly, the levels of PPP intermediates, such as glucose, glucose phosphate, and gluconolactone, were decreased, while DNA precursors, such as uridine and uracil, were increased during the metastatic phase (possibly a consequence of metabolic shunting towards PPP-derived nucleotides, such as ribose, supporting de novo DNA synthesis necessary for lung metastases development) [36,38] (Figure 3). Glutathione pathway downregulation, reduced antioxidant threonic acid levels, decreased arabitol and arabinofuranose levels, and high hypoxia levels also characterize the metastatic phase [38] (Figure 3).

A study of OS highly metastatic human and mouse cell lines also revealed significantly reduced levels of inositol pathway metabolites [38] (Figure 3). Ren et al. explored the effects of inositol pathway dysregulation, exposing metastatic OS cell lines to inositol-6-phosphate, a molecule that is converted to inositol once inside the cell. This exposure led to reduced cellular glycolysis and operated PI3K/AKT signaling downregulation, with suppression of OS metastatic progression. However, the specific mechanisms of inositol-6-phospahate antitumor activity are still not fully disclosed [39].

Supplementing human, canine, and mouse OS cell lines in vitro with the competitive 2-deoxy-D-glucose (2DG) glycolysis inhibitor limited the metastatic phenotype, with an important decrease in cathepsin L (a lysosomal cysteine protease capable of degrading the extracellular matrix), β-actin, and α-tubulin, leading to downregulation of cytoskeletal proteins and reduced invapodial extension length and subsequent decreased cell migration [38].

Recently, Lv et al. collected serum samples from 65 OS patients and compared them with samples from 30 healthy controls [35]. Not only did they identify higher adeosine-5-monophosphate, inosine-5-monophosphate, and guanosine monophosphate serum levels in OS patients compared with healthy controls, but also higher levels of 5-aminopentamide, 13(S)-HpOTrE (FA 18:3 + 2O) and methionine sulfoxide were found in metastatic OS compared with primary OS without metastases [35]. The study authors proposed lactic and glutamic acids as potential diagnostic markers for primary OS, 5-aminopentamide, and 13(S)-HpOTrE (FA 18:3 + 2O) as markers to discriminate metastatic from non-metastatic OS [35].

Cancer stem cells (CSCs) consist of a tiny subpopulation of cancer cells within heterogeneous tumors that are typically aggressive, undifferentiated, with self-renewal capability and ROS molecules sensibility, also showing metabolic hyperactivity [40]. CSC from different tumors show specific energetic and metabolic pathways, even though OXPHOS and glycolysis remain, generally, the primary energy production mechanisms [40]. These cells are able to initiate, propagate, and spread the cancer [40]. CSCs play an important role in refilling the tumor pool, being a precious reservoir of potential distinct differentiated tumor cells [40]. Their immortal nature may contribute to tumor relapse after macroscopic tumor removal [40]. Interesting studies have recently been published regarding OS CSC. La Noce et al. underlined the weight of epigenetic changes as crucial contributive factors to CSC phenotype, showing that the treatment of different OS lines with histone deacetylase (HDAC) 2 inhibitors decreased repressive histone markers, increased active histone markers, increased acetylation, decreased DNA global methylation, thereby inducing an expansion of OS CSC [41]. These findings suggest that HDAC2 may be a potential therapeutic target in human OS [41]. Palorini et al. have shown that 3AB-OS CSC are more dependent on high glycolysis and less dependent on OXPHOS for energy production and survival when compared with OS MG63 cells (non-CSC) [42]. In parallel, 3AB-OS CSC have an augmented expression of lactate dehydrogenase A and a larger accumulation of lactate in the culture medium when compared with OS MG63 cells [42]. Congruously, 3AB-OS CSC exhibited a reduced mitochondrial respiration, a stronger glucose depletion sensitivity, a stronger glycolysis inhibition sensitivity, and a lessened sensitivity to oxidative phosphorylation inhibitors [42].

In the end, it seems clear that OS is associated with metabolic reshaping. Increased levels of metabolites linked with lipid metabolism and amino acid biosynthesis pathways are characteristic [43]. These findings represent the foundations for identifying major targets or biomarkers, capable of aiding in primary diagnosis and metastasis prediction, and for enabling better disease follow-up in the near future.

#### 3.2.2. Chondrosarcoma

Chondrosarcoma (CS), the second most common primary bone tumor, is a cartilage-forming bone neoplasm characterized by hyaline cartilaginous matrix production [44]. Previous studies point towards metabolic adaptations in CS, encompassing glycolysis upregulation and OXPHOS downregulation in high versus low-grade CS [45], hyperactivation of the mTOR pathway with subsequent metabolic adaptations [46], and missense and heterozygous IDH 1/2 mutations leading to 2-HG oncometabolite accumulation [5,47] (Figure 3).

Addie et al. investigated potential key metabolic pathways in CS cell lines, including glycolysis, glutamine metabolism, glutathione, fatty acid metabolism, HIF1α, and mTOR pathways. In the end, the mTOR pathway emerged as the most promising target, with its inhibition showing oxidative and glycolytic metabolism reduction and decreased CS cell line proliferation [48].

IDH is a crucial enzyme that catalyzes the oxidative decarboxylation of isocitrate to α-ketoglutarate and carbon dioxide using NAD+ or NADP+ as cofactors [5]. NADP+-dependent cytosolic isoform IDH1 and mitochondrial isoform IDH2 are significantly homologous [5]. As previously mentioned, IDH 1/2 missense mutations lead to suppression of the IDH ability to convert isocitrate to α-ketoglutarate, endowing IDH with a novel function that consists in reducing, in a NADPH-dependent process, α-ketoglutarate to 2-HG [5]. 2-HG, structurally similar to α-ketoglutarate, is a competitive inhibitor of α-ketoglutarate-dependent-dioxygenases (TETs, JHDMs and PHDs) and its accumulation leads to DNA and histone hypermethylation, with subsequent downregulation of tumor-suppression genes, cellular differentiation blockade, and enhanced tumorigenesis [5]. Further investigation revealed that inhibiting mutant IDH 1/2 significantly decreased 2-HG production, reversed histone and DNA hypermethylation, and promoted cellular differentiation, with AGI-5198 (a specific IDH 1 mutant inhibitor) decreasing 2-HG levels in a dose-dependent manner, as well as significantly inhibiting colony formation and migration in human CS cells [49]. Additionally, IDH 1/2 have defective homologous recombination repair, resulting in sensitivity to poly (ADP-ribose) polymerase (PARP) inhibition, and treatment with the PARP inhibitor olaparib showed clinical benefit in a short series of IDH 1/2-mutated CS patients [50].

#### 3.2.3. Ewing Sarcoma

Ewing Sarcoma (ES) is an aggressive bone or soft tissue tumor most often affecting young patients during childhood and adolescence. Despite significant progress in diagnosis and treatment over the last decades, the room for improvement is enormous, since survival rate for metastatic disease is only 15–20%, despite the 75% reported for localized disease [51]. ES oncogenesis derives from translocation between chromosomes 11 and 22. This event culminates in a fusion product, responsible for merging EWSR1 and FLI1 genes and originating the oncogenic fusion protein known as EWS/FLI1 [52]. This protein plays a key role as an oncogenic transcription factor that misregulates the expression of a significant number of genes.

ES metabolic landscape is poorly characterized. Nonetheless, Tanner et al. reported a metabolic alteration driven by the EWS/FLI1. This chimeric protein induces de novo serine–glycine biosynthesis, nutrients that seem to play a major role in tumor oncogenesis [52] (Figure 3). Additionally, Sen et al. confirmed Tanner findings regarding de novo serine–glycine biosynthesis triggered by EWS/FLI1 [53]. These authors also demonstrated that EWS-FLI1 regulates expression of SLC1A5 amino acid transporter and of two mitochondrial enzymes (MTHFD2 and MTHFD1L) that act in the one-carbon cycle [53]. Recent evidence shows promising results for the combination of PARP and nicotinamide phosphoribosyltransferase (NAMPT) inhibitors (NAMPT inhibitors block the rate-limiting enzyme in production of NAD+, an obligatory substrate of PARP) in depleting NMN and NAD+, decreasing PAR activity, and increasing DNA damage and ES cell apoptosis [54].

#### 3.2.4. Giant Cell Tumor of the Bone

Giant cell tumor of bone (GCT) is a benign bone neoplasm that may present important local aggressiveness and sometimes be misdiagnosed as a bone sarcoma [55]. Despite not being a bone sarcoma, GCT can also originate in lung metastasis in 2–3% of cases, although with much better prognosis compared with metastatic in OS or CS [56,57]. Looking into the GCT metabolomics, Wang et al. reported potential biomarkers provided by GCT metabolic profiles [58]. They found modified glucose, lipid, amino acid, and intestinal microbial metabolisms, with at least 18 metabolites identified as potential biomarkers [58] (Figure 3). However, further validation studies are required to confirm these results.

### 3.3. The Special Case of Gastrointestinal Stromal Tumors

Gastrointestinal stromal tumors (GIST) are mesenchymal tumors mostly attributable to genetic or epigenetic alterations, as KIT and PDGFRα receptors, tyrosine kinase, and SDH subunit mutations [59]. GIST is associated with significant glucose uptake and increased glycolytic activity [60]. Treatment with imatinib promotes decreased glycolytic activity and augmented mitochondrial respiratory capacity in imatinib-sensitive GIST cells, even though this metabolic reprogramming is not observed in imatinib-resistant GIST cells [60]. Early metabolic imatinib responses may be observed in GIST patients through PET using fluorine-18-fluorodeoxyglucose (18FDG), preceding by weeks or months an important tumor size reduction in computer tomography and closely correlating with clinical benefit (namely with symptoms improvement, particularly with pain) [61]. Li et al. showed that GIST maximal standard uptake value (SUVmax) on 18FDG PET-CT correlated with the GIST risk category, tumor diameter, and Ki-67 index in the gastric primary GIST [62]. Furthermore, Albano et al. found an 82% rate of PET avidity rate in these tumors, showing that avidity degree is correlated with stage, tumor risk group, and mitotic index [63].

GIST is therefore a good example of a sarcoma whose metabolic landscape may provides important diagnostic, disease monitoring, and treatment sensitivity information.

## 4. From Biomarkers to Therapeutic Targets

Identifying important metabolites and metabolic pathways in sarcomagenesis led to an increase in biomarkers and potential therapeutic target numbers. Concurrently, other targeted therapies exert their effect on sarcoma growth and proliferation by directly or indirectly modulating the metabolome of different sarcoma types. Herein will be briefly addressed some additional pathways whose deregulation shapes sarcoma metabolome, contributing to sarcomagenesis, and respective available targeted therapies.

### 4.1. mTOR Signaling Pathway Inhibition

The Pi3k/Akt/mTOR pathway directly controls protein and lipid synthesis, autophagy, and glucose metabolism [64,65]. mTOR is composed of two distinct multiprotein complexes, mTORC1 and mTORC2 [65]. These proteins act as regulators of cellular metabolic homeostasis. mTORC1 inhibition leads to negative regulation of ribosomal protein S6 (S6) phosphorylation state, with subsequent reduction in energy (ATP) and cofactor (NADPH) generation, both essential for glucose metabolism and other biosynthetic processes, compromising cell survival and proliferation [66].

mTOR inhibitors already being investigated in sarcomas include rapamycin, temsirolimus [67], everolimus [68,69], and ridaforolimus [70] (Table 2). In sarcoma tumor models, rapamycin significantly reduced tumor volume compared to placebo [71,72]. Depending on the model used, differences in treatment effectiveness were observed, since the more dependent the tumor is on glycolysis, the more sensitive it is to rapamycin-induced growth inhibition [72]. Additionally, glycolytic flow decrease induced by rapamycin use in vivo may activate the pro-apoptotic pathway, as shown by increased caspase-3 staining [72]. Nevertheless, the relationship between the glycolytic status and apoptosis induction is still not well understood [72].

### 4.2. β-Catenin Gene Mutation Modulation

Using broad-spectrum metabolomics, differences were explored between paired normal fibroblasts and desmoid tumor cells from patients with desmoid tumor diagnosis [73]. Desmoid tumors are locally invasive soft tissue tumors that lack the ability to metastasize, the majority of which are related to T41A and S45F mutations on the beta-catenin encoding gene (CTNNB1) [73]. Desmoid tumors are the paradigm for dasatinib and FAK inhibitor 14 treatment [73] (Table 2). Despite differences in the metabolomic profile of the two beta-catenin mutations, T41A and S45F, administration of dasatinib and FAK inhibitor 14 resulted in a reshaped metabolic profile, both in normal fibroblasts and in desmoid tumor cells, with the cell line differentiation process led by aminoacyl-tRNA biosynthesis in mitochondria and cytoplasm, and by signal transduction amino acid-dependent mTORC1 activation [73]. This study offered the first insight into differences in the metabolome of paired normal and desmoid tumor cells and how these tumor cells respond to desmoid tumor therapeutics, highlighting new target pathways [73].

### 4.3. BCR-ABL and Src Signaling Inhibition

Analysis of patient samples indicates the frequent involvement of diverse point mutations in the BCR-ABL kinase domain, which render it unable to bind to STI571 and lead to development of increased BCR-ABL copy numbers [74,75]. The main molecule targeting BCR-ABL tyrosine kinase domain is imatinib, but other tyrosine kinases inhibitors are available, as dasatinib [76] (Table 2). Dasatinib is a targeted agent that inhibits multiple tyrosine kinases, including Src, BCR-ABL, c-Kit, PDGFRβ, and FGFR-1, with important redefining effects on tumor metabolomic landscape [75].

Dasatinib was originally labelled as a Src kinase inhibitor and later shown to also inhibit BCR-ABL. Recently, dasatinib was shown to inhibit Src and downstream FAK signaling at nanomolar concentrations, blocking cell migration and invasion in several human sarcoma cell lines [77,78]. It seems to be an apoptotic inducer in bone sarcoma cells [77,78]. Furthermore, Src expression knockdown by small interfering RNA (siRNA) in bone sarcoma cells also induces apoptosis, suggesting that the observed dasatinib response in these cells is conveyed through Src-mediated signaling inhibition [79]. Together, these findings indicate that dasatinib is a promising therapeutic agent for preventing growth and metastasis in a wide diversity of soft tissue and bone sarcomas. Other tyrosine kinase inhibitors, such as pazopanib (PDGFRα, PDGFRβ, and VEGFR inhibitor) and olaratumab (PDGFRα inhibitor), are already approved for sarcoma treatment [80], but their metabolic effects are not widely explored (Table 2).

### 4.4. PARP and Nicotinamide Phosphoribosyltransferase Activity Inhibition

PARP is a large family of enzymes involved in several cellular processes, including DNA single-strand break repair [81]. PARP inhibitors (PARPi) exert antitumor activity by both catalytic PARP inhibition and PARP–DNA trapping, and represent a potential synthetic lethal approach against cancer cells with specific DNA-repair defects [81].

Pharmacological inhibition of nicotinamide phosphoribosyltransferase (NAMPT) almost invariably leads to intracellular NAD+ depletion and, when protracted, to ATP shortage and cell demise [82].

Cancer cells and activated immune cells express high nicotinamide phosphoribosyltransferase (NAMPT) levels and are highly susceptible to NAMPT inhibitors (NAMPTi), as shown by activity of these agents in malignant disorder models [82].

Preclinical and clinical studies showed promising results in sarcoma, with the most robust PARPi efficacy evidence obtained in Ewing sarcomas bearing EWS–FLI1 or EWS–ERG genomic fusions [81].

PARPis have emerged as a treatment strategy for patients with Ewing sarcoma, but in preclinical in vivo models and clinical trials PARPis have failed to demonstrate meaningful response in Ewing sarcoma patients [54]. Combining PARPis with NAMPTis blocks the rate-limiting step in NAD+ production, enhancing PARP inhibition without additive toxicity. This synergy showed robust in vitro results in Ewing sarcoma, through decreased PAR activity, increased DNA damage, and apoptosis, and retained efficacy in multiple in vivo models, showing its potential for use in Ewing sarcoma patients [54] (Table 2).

PARP inhibitor activity in sarcoma also seems to be enhanced by chemotherapy and radiation [81]. Its use in advanced-stage STSs, alone or combined in multimodal treatments, is of great interest [81].

### 4.5. MicroRNAs (miRNA) Inhibition

MicroRNAs (miRNAs) are small non-protein-coding RNA molecules that exert regulatory functions on gene expression [83,84].

In Oncology, namely in sarcomas, miRNAs may have screening, diagnostic, prognostic, and predictive significance and be used as therapeutic targets [84,85] (Table 2).

Using a microarray approach, miRNA expression profiles were characterized in a series of 27 sarcomas from seven different histological types. Four major groups were identified based on miRNA expression patterns, with three groups predominantly consisting of the same tumor types: synovial sarcomas, leiomyosarcomas, and GIST [86].

In Ewing sarcoma, several studies have implicated miRNAs in pathogenesis, from disease development to metastasis formation. miRNAs have opened a novel field in sarcoma research [87].

### 4.6. Isolated Proteasome Inhibition and Combined Proteasome and Histone Deacetylases (HDAC) Inhibition

The ubiquitin–proteasome pathway is key in cellular homeostasis, being responsible for the removal of damaged, misfolded or deleterious proteins from the cellular environment [88]. By blocking this pathway, toxic proteins accumulate inside the cell, ultimately leading to apoptosis and cell death [89]. Efficacy of this therapeutic modality is dependent on cell protein turnover, with the higher the better [89].

Although proteasome inhibitor efficacy has been mainly studied and demonstrated in different hematological malignancies with high IgG production [89], its use has also been investigated in the sarcoma setting.

A class of benzyl-4-piperidone compounds disrupt 19S proteasome function through inhibition of USP14 and UCHL5 deubiquitinating enzymes, selectively inhibiting growth of Ewing sarcoma cell lines and inducing their apoptosis [90]. The proteasome inhibitor bortezomib was shown to induce apoptosis on Ewing sarcoma cell lines [91] (Table 2). The combined use of the HDAC inhibitor quinostat and a proteasome inhibitor suppressed tumor growth in a synovial sarcoma murine model (Table 2). Quinostat disrupts the SS18-SSX driving protein complex, reestablishing expression of EGR1 and CKN2A tumor suppressors, and its combination with a proteasome inhibitor additionally inhibits the aggresome formation in response to proteasome inhibition, leading to elevated endoplasmic reticulum stress, activation of BIM and BIK pro-apoptotic effector proteins, BCL-2 phosphorylation, and increased ROS levels [92].

### 4.7. Immune Checkpoint Inhibition

The immune system physiologically destroys non-self-cells, leaving self-ones intact [93]. Keeping an appropriate balance between immune cell activation and deactivation is crucial, since immune cell constitutive activation may lead to the destruction of healthy cells [93]. T cells are usually inactivated by the action of an “off switch” group of proteins called immune checkpoints [93]. Immune checkpoint inhibitors block the link between immune checkpoints and their partner proteins, allowing constitutive activation of T cells and subsequent immune-mediated destruction of specific cells, like cancer clones [93]. A group of biomarkers, comprising tumor-infiltrating lymphocytes, PD-1 and PD-L1 expression, mutational load, and DNA mismatch repair deficiency, have been used as barometers of sarcoma responsiveness to ICI [94].

It remains unclear which sarcoma patients may benefit from immune checkpoint inhibition (and subsequent TME immune component and cancer cell metabolome modulation), with UPS, a subtype with higher mutational burden, higher T-cell fraction, and higher PD-1 and PD-L1 levels, and show promising response rates to pembrolizumab [95] (Table 2). OS also displays high antigen and neoantigen burden, which confers immunogenic potential to this sarcoma subtype [96]. On the other hand, synovial sarcoma and round-cell/mixed liposarcoma have an immunologically quiet TME and lower sensitivity to anti-PD1 therapy [95]. Combination therapies are now being investigated, including pembrolizumab with axitinib, a tyrosine kinase inhibitor, encouraging T-cell trafficking into TME [95] (Table 2). Gemcitabine modulates vasculature, besides having direct cytotoxic effects, and is also being tested in combination with pembrolizumab [95] (Table 2).

## 5. Conclusions

Sarcoma metabolomics is a broadly unexplored field that can offer diverse opportunities. Deeper characterization and a sharper picture of sarcoma metabolic and microenvironment landscape may pave the way for diagnostic and staging refinement and identification of new potential therapeutic targets, resulting in benefits for patients.

## Figures and Tables

**Table 1:** Cancer metabolic adaptations and acquired phenotypes.

Metabolic Hallmark	Alterations and Adaptations in Cancer	Outcome/Acquired Phenotype
Deregulated uptake of glucose and amino acids [5]	(1) Mutations of the oncogenes c-MYC, KRAS and YAP [8] (2) Overexpression of YAP and loss-of-function mutations in p53 [8] (3) Phosphoinositide 3-kinase (PI3K)/Akt pathway hyperactivation [5,8] (4) C-MYC, n-MYC, mTORC1, IL-4 and lactate modulation [8] (5) RAS mutations [8]	(1) Upregulate glucose transporter (GLUT) 1 expression [8] (2) Augments GLUT3 expression [8] (1) and (2) Increase entrance of glucose into the cell [8] (3) Promotes GLUT1 mRNA expression and GLUT1 protein translocation from the inner membranes to the cell surface [5] and hexokinase (HK)2 activity upregulation, trapping glucose inside the cell [8] (4) Upregulates ASCT2 glutamine transporter expression increasing entrance of glutamine into the cell [8] (5) Increases glutamine uptake by micropinocytosis [8]
Use of opportunistic modes of nutrient acquisition [5]	(1) Hypoxia triggers the expression of transcription factors called hypoxia-inducible factors (HIF) [9] (2) Cholesterol depletion induces activation of sterol regulatory element-binding proteins [9] (3) Amino acid deprivation leads to activation of the GCN2 kinase [9] (4) Ras or c-Src mutations [5] (5) Prolonged periods of extracellular nutrients absence lead to macroautophagy [9]	(1) Stimulates glucose uptake, lactate export, glycolysis and angiogenesis (by induction of VEGF expression) [9] (2) Stimulates the expression of enzymes required for de novo synthesis of fatty acid and sterol lipids, increases LDL receptors expression and enhances NADPH production [9] (3) Promotes selective translation of mRNAs like ATF4, promoting the transcription of amino acids transporters and enzymes involved in the generation of non-essential amino acids [9] (4) Enhances the recovery of free amino acids by lysosomal digestion of extracellular proteins by several processes including micropinocytosis, degradation of entire living cells (entosis) and digestion of apoptotic cellular corpses [5] (5) Sequestrates and promotes lysosomal digestion of cytosolic macromolecules and organelles, allowing the recycling of these cellular components into nutrients [9]
Use of glycolysis/TCA cycle intermediates for biosynthesis and NADPH production [5]	(1) C-MYC and β-catenin/TCF signaling hyperactivation [5]	(1) Leads to overexpression of multiple key enzymes for generation of diverse glycolytic and TCA cycle intermediates that are biosynthetic precursors [5]
Increased demand for nitrogen [5]	(1) C-MYC signaling hyperactivation [5] (2) Asparagine synthetase upregulation [5] (3) Glutamine synthetase upregulation [5]	(1) Promotes celular glutamine uptake, upregulates the expression of different enzymes with roles in nucleotide biosynthesis and upregulates glutaminase [5] (2) Increases asparagine synthesis (crucial in glutamine deprived conditions) [5] (3) Augments intracelular de novo glutamine production (fundamental in glutamine deprived conditions) [5]
Alterations in metabolite-driven gene regulation [5]	(1) Diverse oncogenic pathways hyperactivation [10] (2) Loss-of-function SDH and FH mutations [10] (3) Gain-of-function IDH1 and IDH2 mutations [10]	(1) Enhances total histone acetylation, leading to increased and broader oncogene expression [10] (2) Succinate and fumarate accumulation leads to inhibition of demethylases (JmJC and TET), increase of genome wide DNA and histone hypermethylation, enabling oncogenic promoter-enhancer interactions, inducing epithelial-to-mesenchymal transition, and disrupting DNA repair mechanisms [10] (3) Catalyzes the conversion of α-ketoglutarate to 2-HG, leading to 2-HG accumulation, DNA and histone hypermethylation with downregulation of genes associated with tumor-suppression and cellular differentiation blockade [10]
Metabolic interactions with the microenvironment [5]	(1) Low glucose and aminoacids (glutamine, L-arginine, methionine) extracellular availability and extracellular lactate accumulation [11] (2) Increased CAF glycolytic and glutamine anabolic metabolism [11] (3) CAF-derived exosomes proliferation [11] (4) Metabolic plasticity (glycolysis vs. mitochondrial metabolism) relative to local oxygen availability [11]	(1) Decreases mTOR activity leading to an impairment of T cell (CD8+) and NK cell function and proliferation and promotes a macrophage M2 polarization [11] (2) Leads to use of resultant metabolites from CAF glycolysis and glutamine metabolism to fuel cancer cells [11] (3) Supplies cancer cells with amino acids, lipids and TCA intermediates [11] (4) Sustains glucose consumption, glycolysis and OXPHOS in cancer cells located in well perfused areas, while cells in poorly perfused areas depend on other carbon sources [11]

GLUT1—Glucose Transporter 1; GLUT3—Glucose Transporter 3; PI3K/Akt—Phosphoinositide 3-kinase/Protein kinase B; HK2 Hexokinase 2; ASCT2—Alanine, Serine, Cysteine Transporter 2; HIF—Hypoxia inducible-factors; VEGF—Vascular Endothelial Growth Factor; LDL—Low-density lipoprotein; NADPH—Nicotinamide adenine dinucleotide phosphate; GCN2—General control nonderepressible 2; ATF4—Activating transcription factor 4; SDH—Succinate dehydrogenase; FH—Fumarate hydratase; JmJC—Jumonji C; TET—Ten eleven translocation methylcytosine dioxygenases; IDH—Isocitrate dehydrogenase; 2-HG—2-hydroxyglutarate; mTOR—Mechanistic target of rapamycin; NK—Natural killer; CAF—Cancer associated fibroblasts; TCA—Tricarboxylic acid cycle; OXPHOS—Oxidative phosphorylation.

**Table 2:** Deregulated metabolic pathways and respective therapeutic targets.

Therapeutic Target	Alterations and Adaptations in Cancer
mTOR signalling pathway inhibition	Rapamycin, Temsirolimus, Everolimus and Ridaforolimus
Beta-catenin gene mutations modulation	Dasatinib and FAK inhibitor 14
BCR-ABL and Src signalling inhibition	Imatinib, Dasatinib, Pazopanib and Olaratumab
PARP and NAMPT activity inhibition	PARP inhibitors and NAMPT inhibitors
miRNAs inhibition	miRNAs
Proteosome and HDAC inhibition	Bortezomib and Quinostat
Immune checkpoint inhibition	Pembrolizumab (monotherapy or combined with Axitinib or Gemcitabine)

mTOR—Mechanistic target of rapamycin; FAK—Focal adhesion kinase; PARP—poly (ADP-ribose) polymerase; NAMPT—Nicotinamide phosphoribosyltransferase; miRNA—microRNAs; HDAC—Histone deactylase.
